# What are the Benefits of Interacting with Nature?

**DOI:** 10.3390/ijerph10030913

**Published:** 2013-03-06

**Authors:** Lucy E. Keniger, Kevin J. Gaston, Katherine N. Irvine, Richard A. Fuller

**Affiliations:** 1 School of Biological Sciences, University of Queensland, Brisbane, Queensland 4072, Australia; E-Mail: r.fuller@uq.edu.au; 2 Environment and Sustainability Institute, University of Exeter, Penryn, Cornwall TR10 9EZ, UK; E-Mail: k.j.gaston@exeter.ac.uk; 3 Institute of Energy and Sustainable Development, De Montfort University, The Gateway, Leicester LE1 9BH, UK; E-Mail: kirvine@dmu.ac.uk or kirvine@umich.edu

**Keywords:** urbanisation, ecosystem services, health benefits

## Abstract

There is mounting empirical evidence that interacting with nature delivers measurable benefits to people. Reviews of this topic have generally focused on a specific type of benefit, been limited to a single discipline, or covered the benefits delivered from a particular type of interaction. Here we construct novel typologies of the settings, interactions and potential benefits of people-nature experiences, and use these to organise an assessment of the benefits of interacting with nature. We discover that evidence for the benefits of interacting with nature is geographically biased towards high latitudes and Western societies, potentially contributing to a focus on certain types of settings and benefits. Social scientists have been the most active researchers in this field. Contributions from ecologists are few in number, perhaps hindering the identification of key ecological features of the natural environment that deliver human benefits. Although many types of benefits have been studied, benefits to physical health, cognitive performance and psychological well-being have received much more attention than the social or spiritual benefits of interacting with nature, despite the potential for important consequences arising from the latter. The evidence for most benefits is correlational, and although there are several experimental studies, little as yet is known about the mechanisms that are important for delivering these benefits. For example, we do not know which characteristics of natural settings (e.g., biodiversity, level of disturbance, proximity, accessibility) are most important for triggering a beneficial interaction, and how these characteristics vary in importance among cultures, geographic regions and socio-economic groups. These are key directions for future research if we are to design landscapes that promote high quality interactions between people and nature in a rapidly urbanising world.

## 1. Introduction

Throughout history, humans have had an intimate relationship with nature, most obviously depending on it for subsistence and production. As modern society emerged, and the human population condensed into urban areas, industrialisation freed many people from reliance on direct consumptive interactions with nature. Indeed, in post-war society, people-nature interactions have fundamentally shifted from direct consumption and exploitation to more mutualistic relationships in which people actively seek out interactions with nature for recreation and enjoyment [1,2]. Interacting with nature may therefore be important not only for survival, but also for human quality of life [3,4,5]. Indeed, there is mounting empirical evidence that interacting with nature delivers a range of measurable human benefits [6,7,8], including positive effects on physical health [9,10,11], psychological well-being [12,13,14], cognitive ability [15] and social cohesion [16]. Reviews on this topic have been published in the past, but these have generally focused on a specific type of benefit, e.g., [17], have been limited to a single discipline, e.g., [18,19,20], or have covered the benefits arising from a particular type of interaction, e.g., [21,22,23].

Understanding the benefits of interacting with nature is important for maintaining and improving human well-being in a rapidly urbanising world. For example, evidence that living in close proximity to green spaces delivers health benefits [24] could be used to design landscapes with broader societal benefits such as reductions in health spending [18] or crime rates [25]. However, without a clear view of the quality of evidence, and information on any potential thematic or geographic bias in the available information, it is difficult to come to clear conclusions about which particular features of the environment, including their ecological characteristics, might deliver these benefits.

Our review focuses on three critical questions. First, we explore the geographic distribution of studies into the benefits of interacting with nature. This is an important issue, not least because the amount and type of nature that can be experienced varies enormously across the World. For example, the tropics are much more biodiverse than higher latitudes [26] and the extent to which natural landscapes have been cleared for human use also shows a strong spatial pattern [27]. Important changes in the type of biodiversity present accompany the increase in biodiversity toward the tropics. For example, bites from venomous snakes show a very strong bias toward equatorial regions [28], and zoonotic diseases are similarly geographically biased [29], suggesting that some of the more important negative aspects of interacting with nature might be overlooked if the research is biased toward high latitudes. Bias in the location of studies could also lead to skewed representation of different human cultures [30,31].

Second, we examine the types of benefits studied to understand whether some have received more focus than others. For example, the growing availability of databases of health status or interventions and detailed land maps has facilitated correlational investigation of access to green space with health outcomes such as morbidity, e.g., [10,24]. In contrast, less tangible benefits such as spiritual well-being or social cohesion may be more challenging to study.

Third, we review in some detail the basis for the evidence surrounding each of the identified benefits of people-nature interactions. For a benefit to happen, a setting in which nature can be experienced must exist, and an interaction between humans and nature must occur. However, we are aware of no published typologies of these settings, and few that detail the interactions [7,23,32] or benefits [7,33], which hinders a synthesis of the research into the benefits of experiencing nature. Before going on to address the three questions outlined above, we first build typologies of: (i) the settings in which people-nature interactions can occur, (ii) the types of interactions that occur between people and nature, and (iii) the benefits that can arise from these interactions.

## 2. Methods

### 2.1. Selection of Articles for Review

We took a broadly qualitative approach to our review, rather than conducting a formal meta-analysis. Our survey of the literature indicated that much of it is not amenable to quantitative meta-analysis, and that such an approach would necessitate filtering out all but a small proportion of studies. We restricted our search to primary research articles in the peer-reviewed scientific literature, identifying articles relevant to this review through standardised search methods including electronic database searches (Web of Science, Google Scholar) and opportunistic searches through relevant reference lists. Search terms were combinations of “nature”, “interaction”, “benefit”, “health” and “biodiversity”. Studies were included if they presented findings about one or more benefits of interacting with nature in some form. Studies were excluded if they reported the benefits of nature for humans but did not focus on a specific interaction at an individual level, given that our focus was on interactions. For example, a study reporting on the economic and environmental benefits of biodiversity [34] was excluded because it did not directly investigate a specific interaction between people and nature. Only studies published in peer-reviewed scientific journals were included, and our search was conducted in June 2011. The search yielded 57 papers for review.

### 2.2. Typology of Settings

We categorised the settings in which the reviewed research had been done into six types, loosely arranged in increasing order of naturalness of the environment. These settings ranged from indoor spaces, through outdoor local to broad regional landscapes (Table 1). Indoor settings were either private (e.g., within a home) or in a public building such as a hospital, office or classroom. The natural elements present in these settings were typically indoor plants, pictures of nature, or window views of nature. Urban outdoor settings were characterised by a high density of houses, traffic and people, but include some green spaces and natural elements such as public and private parks, gardens, landscaping and street trees. These settings differed from those on the urban fringe, where housing density tends to be lower and large natural areas such as forest reserves are more prevalent. Production landscapes were intensively managed for agriculture, characterised by open spaces, large areas of vegetation and broad landscapes with few buildings and little traffic. Wilderness settings were largely undisturbed by people and generally remote from dense urban centres; apart from the few people residing in such landscapes, specific trips to experience them would normally be necessary. We also included a species-specific setting because some of the reviewed papers identified benefits associated with interacting with a specific species, regardless of where that interaction occurred.

**Table 1:** Typology of settings in which interactions between people and nature occur.

Setting	Description	Examples
Indoor	Inside a building	Foliage plants [35,36]
Urban	Landscape dominated by built form	Public green space [37]
		Private green space e.g., garden [38]
		Roadside trees or isolated urban vegetation [39]
Fringe	The area immediately surrounding a town or city	Peri-urban nature reserve [37]
Production Landscape	Agricultural lands (pastoral or cropping)	Paddocks/fields/countryside [10]
Wilderness	Area where human influence is low	Beach [40]
		Ocean [41]
		River [42,43]
		Water [44,45]
		Mountains [46]
		Forest/woodland [47]
		National Parks [48,49,50]
Specific species	Cases where object of the interaction is defined with no particular setting	Marine animals [41,51]
		Avian [41]
		Domesticated pets [52]

### 2.3. Typology of Interactions

Three main types of interaction were evident from a review of the selected literature: indirect, incidental and intentional (Table 2). Indirect interactions do not require a person to be physically present in nature, and can include such activities as viewing an image or motion picture of nature, or having a view of nature through a window. Incidental interactions occur when a person is physically present in nature, but where the interaction is an unintended result of another activity, such as encountering vegetation whilst cycling to work. Incidental interactions differ from indirect interactions in that nature, or natural elements, must be physically present. For example, viewing a picture of a plant inside an office building is an indirect interaction, whereas encountering the plant is an incidental interaction because the plant is physically present in the same space as the person. Intentional interactions are those in which the participant has intent to interact with nature, such as viewing wildlife, gardening or hiking in a national park. Note that in some special cases where a window is used to conceal a wildlife-watcher, viewing nature through a window can be thought of as an intentional interaction, e.g., watching a bird feeder that has been set up in a backyard or viewing wildlife from a specially designed hide. Researchers have previously thought about interactions as a continuum ranging from passive to active [7,8,23], and while very useful, such schemes do not account for the personal intent behind an interaction. This is an important distinction because it seems likely that only a certain proportion of a population will engage intentionally with nature [53,54], and it has been argued that the intent to interact may be critical in efforts to promote sustainable behaviours [55,56].

**Table 2:** Typology of interactions between people and nature.

Interaction	Description	Examples
Indirect	Experiencing nature while not being physically present in it	Viewing nature in a picture, image, motion picture or through a window [13,57]
Incidental	Experiencing nature as a by-product of another activity	Encountering nature incidental to another activity, e.g., walking to work or driving [39]
		Encountering vegetation indoors [58]
Intentional	Experiencing or being in nature through direct intention	Recreation, e.g., hiking, camping, wildlife viewing, adventure
		Gardening or farming [16]
		Conservation volunteering [47]

### 2.4. Typology of Benefits

An enormous range of benefits from interacting with nature has been studied in the reviewed literature. These span from physical health and cognitive benefits to spiritual benefits and the tangible outcomes associated with food production and wealth (Table 3). While we do not focus on the negative aspects of interacting with nature, we recognise their importance for understanding the full scope of the people-nature relationship [29,30,59].

**Table 3:** Typology of the benefits of interacting with nature.

Benefit	Description	Examples
Psychological well-being	Positive effect on mental processes	Increased self-esteem [32,60,61]
		Improved mood [58,32]
		Reduced anger/frustration [62]
		Psychological well-being [13,63,64]
		Reduced anxiety [65]
		Improved behaviour [15]
Cognitive	Positive effect on cognitive ability or function	Attentional restoration [12,14,46,66,67]
		Reduced mental fatigue [63]
		Improved academic performance [68]
		Education/learning opportunities [49,55]
		Improved ability to perform tasks [15]
		Improved cognitive function in children [69]
		Improved productivity [35,68]
Physiological	Positive effect on physical function and/or physical health	Stress reduction [37,70,71]
		Reduced blood pressure [45,32]
		Reduced cortisol levels [70]
		Reduced headaches [37]
		Reduced mortality rates from circulatory disease [24]
		Faster healing [9]
		Addiction recovery [43]
		Perceived health/well-being [59]
		Reduced cardiovascular, respiratory disease and long-term illness [11]
		Reduced occurrence of illness [15,35]
Social	Positive social effect at an individual, community or national scale	Facilitated social interaction [72,73]
		Enables social empowerment [62,74]
		Reduced crime rates [25]
		Reduced violence [63]
		Enables interracial interaction [16]
		Social cohesion [72]
		Social support [72]
Spiritual	Positive effect on individual religious pursuits or spiritual well being	Increased inspiration [42]
		Increased spiritual well-being [41,47]
Tangible	Material goods that an individual can accrue for wealth or possession	Food supply [38]
		Money [50,75]

## 3. Results and Discussion

### 3.1. Geographical Bias

**Figure 1:**
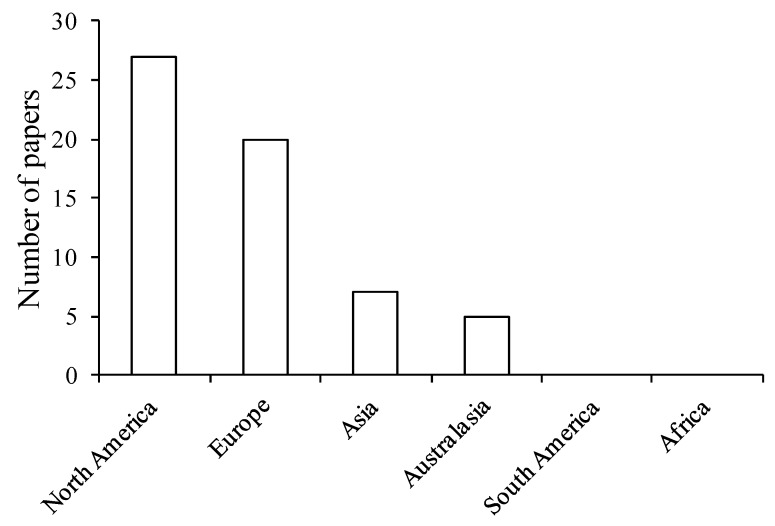
Number of studies undertaken in each continent (n = 59). Where a study investigated more than geographical location, each location is separately included.

The 57 studies were heavily biased toward high latitudes, in particular North America and Europe, with 79% of papers reporting results from these regions (Figure 1). Asia and Australasia were less well represented, and no studies were located in South America or Africa. This bias towards Western, developed nations may be affecting the intensity with which different types of benefits have been studied to date, in part because the distribution of biodiversity is strongly spatially structured, and because cultural and socio-economic differences between regions may influence responses to interactions with nature [30,31,76]. The bias away from heavily biodiverse tropical regions might also result in a heavy focus on the benefits and not the problems provided by nature (e.g., disease vectors, venomous animals).

### 3.2. Disciplinary Representation in Nature-Interaction Benefits Research

**Figure 2:**
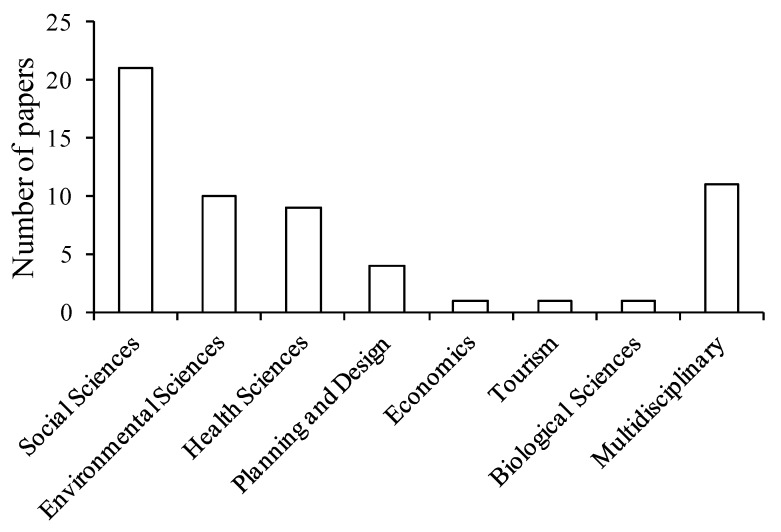
Number of studies undertaken in each research discipline (n = 57). The research discipline for each of the reviewed papers was defined using the classification system employed by the Australian Research Council (2010).

The reviewed studies were published in journals from a broad range of research disciplines (Figure 2). Social science was the most represented discipline; health and environmental sciences were much less heavily represented and only one of the reviewed studies was published from the biological sciences. This bias towards the social sciences and clear deficiency of input from the biological sciences may be constraining understanding of the beneficial interactions between people and nature. Without collaboration from biologists and ecologists, it will be difficult to evaluate the importance of biodiversity and ecological quality of natural environments as mechanisms behind these beneficial interactions.

### 3.3. Strength of Evidence for the Benefits of Interacting with Nature

**Figure 3:**
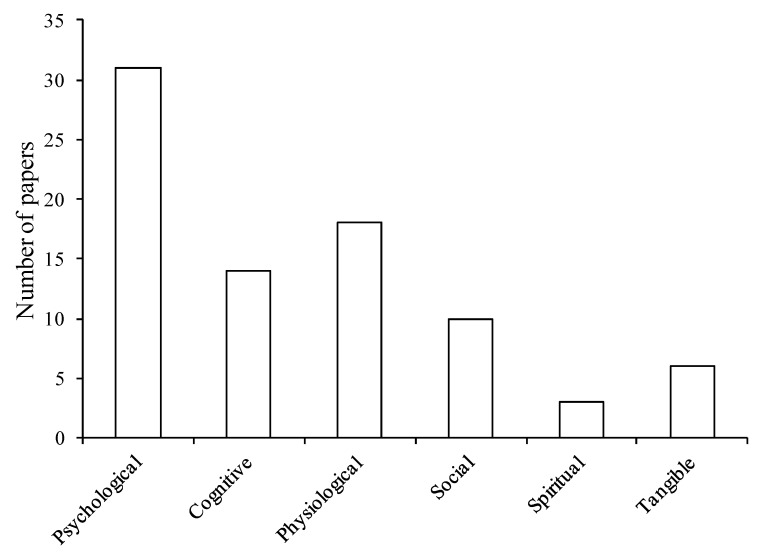
Number of studies that have investigated each type of benefit (n = 82). Where a study investigated more than one benefit, each is separately included.

The majority of studies (Figure 3) focused on psychological benefits, which can broadly be divided into psychological well-being benefits and those associated with cognitive performance. Interaction with nature can increase self-esteem and mood [32,38,62], reduce anger [63], and improve general psychological well-being with positive effects on emotions and behavior [13,64]. These interactions can also have positive effects on cognitive function such as academic performance [68] and the ability to perform mentally challenging tasks [15]. Additionally, this review suggests that interactions with nature may have physical health benefits such as stress reduction [35,70,71] or reduced mortality rates [24] as well as social, including facilitating social interaction [16,72] or reducing crime and violence in urban areas [25,63]. Only a small number of studies focused on spiritual or tangible benefits (Figure 3). We now turn to an evaluation of the strength of evidence supporting each of the major benefits of interacting with nature.

#### 3.3.1. Psychological Well-Being Benefits

Several of the reviewed studies have focused on psychological well-being effects arising from exercising in a natural environment. In a study investigating the effects of green exercise initiatives in the UK, Pretty *et al.* [77] found that participants’ mood and self-esteem, as measured by self-report surveys, were significantly improved after exercise. The authors interpreted this as evidence that exercise in natural environments can improve psychological well-being, although a control treatment with exercise in a non-natural setting was not included, and so it is not clear whether the natural environment itself was contributing to the effect, or whether exercise alone is sufficient. However, in a separate but topically related study, Pretty *et al.* [32] compared the emotional responses of participants exposed to a sequence of natural and urban landscape images whilst running on a treadmill in a laboratory. Mood and self-esteem both improved with exposure to natural scenes, suggesting that exercise may deliver greater benefits when it occurs in the presence of nature. 

Despite the results of these studies, several papers report little or no relationship between the setting in which exercise takes place and psychological well-being benefit. A study in Japan monitored emotional change after exercise in a laboratory and in the green space around a university campus [78]. Positive emotions were found to be significantly higher after exercise, regardless of the running environment [78]. In a related study comparing the effects of exercise in a park and an urban setting in Sweden, Bodin and Hartig [12] found that anxiety, depression and anger, measured using self-report questionnaires, were significantly reduced after running, but there was no significant difference between the running environments. Consequently, we cannot yet conclude definitively that a natural setting increases the psychological well-being benefits of exercise.

Intentional interactions with nature such as gardening [64,70] and watching wildlife [41] also promote psychological well-being. Similarly, interacting with nature may have specific psychological well-being benefits for children. In a study on children’s mental and social health, Maller [62] found that direct contact with nature, as facilitated by curriculum-based nature activities in schools, had a positive impact on self-esteem and mental-well-being. These studies rely primarily on data derived from the perceptions of parents and teachers; experimental research designs that that utilize objective assessments of the psychological well-being effects of interacting with nature on children in a series of controlled environments may be useful for further investigating these effects. Despite the limitations of the reviewed studies, it is clear that interacting with nature may deliver several positive psychological well-being benefits to children.

In addition, there is emerging evidence that childhood interactions with nature may influence attitudes towards nature in later life [79]. While this is not necessarily a benefit *per se*, there is much interest from a sustainability perspective in how attitudes and behaviours that are positive toward nature develop [56,80,81]. A nationwide survey of US adults currently living in urban areas found that growing up in natural environments, or participating in activities such as gardening, visiting parks and taking environmental classes during childhood had a strong influence on positive environmental attitudes in adult life [78]. This was an important study as it highlighted the potential for long-term effects of experiences with nature during childhood. Chawla [82] reviewed several studies in this area, noting that they are limited by survey response bias, sampling bias and the lack of adequate control groups. Long-term longitudinal studies of children living in both urban and natural settings are necessary to determine the importance of these interactions for triggering environmentally responsible behaviour in later life. These studies are of particular importance given that a majority of the human population is now living within urban areas [4,83], where opportunities for people to interact and bond with nature are greatly diminished. Children raised in nature-deficient areas may be deprived of these early interactions with nature and may therefore be less likely to appreciate and support conservation initiatives in adulthood [84]. There are several validated tools available for measuring nature appreciation, including the Connectedness to Nature Scale [85], the Nature Relatedness Scale [86] and the New Ecological Paradigm [87], and, as well as being used to track the development of nature appreciation, they can be used to test whether nature-oriented participants are more likely to derive benefits from interactions with nature than those with less prior affinity to nature.

#### 3.3.2. Cognitive Benefits

It has been hypothesised that natural environments are restorative, contributing to attentional recovery [88] and reducing mental fatigue. Kaplan [13] investigated the restorative benefits of the view from home by comparing window views in six low-rise apartment communities in Michigan (USA). Participants completed self-report questionnaires to measure satisfaction with their neighbourhood and well-being outcomes associated with attentional recovery and mental fatigue; participants also indicated the natural content of the view from their apartment windows. The study found that satisfaction and several well-being outcomes increased with the natural content in the view from home. 

Natural environments vary in their complexity and species diversity and Han [67] found that natural environments also differ in their restorative potential. Importantly, this suggests that general comparisons between natural and urban settings may be deficient because they do not factor in the complexity of different natural environments. In a study from the UK, Fuller *et al.* [14] compared 15 urban green spaces and found that the restorative benefits to park users, as measured by self-report ability to reflect (a dimension of Attention Restoration Theory), increased with plant species richness in the green spaces. This highlights the importance of considering biological diversity and complexity when investigating the benefits of interacting with nature; this study was the only reviewed paper published from the biological sciences discipline, and the only study that specifically investigated the benefits of interacting with biodiversity, as opposed to an often loosely defined “nature”.

Urban environments can be very stimulating, requiring directed attention and negatively impacting cognitive function [88,89]. In urban areas, attention must be directed towards avoiding potential hazards and coping with noise and visual stimuli. This directed attention requires effort in order to inhibit or address these distractions and this can lead to mental fatigue, resulting in a lowered ability to concentrate and perform cognitive tasks [66]. There is good evidence to suggest that exposure to nature in urban and wilderness settings can improve cognitive function and performance [46,89].

Most studies of cognitive benefits used controlled experiments to measure cognitive performance. In a study of 38 students in Michigan (USA), Berman *et al.* [89] measured cognitive performance with a backwards digit span task, in which participants listen to a sequence of numbers and repeat them in reverse order. The results showed that cognitive performance was greater after students had walked through a tree lined arboretum when compared with a busy city street. The authors interpret this as evidence that the restorative properties of nature can improve cognitive function. Similarly, Hartig *et al.* [46] compared wilderness and non-wilderness vacations in southern California (USA) and found that proofreading, as a measure of cognitive performance, was significantly improved in the wilderness vacation participants for several weeks after the vacation, whereas the other two groups declined in proofreading performance over time. The authors conclude that a prolonged wilderness experience has restorative effects [46].

The restorative benefits of nature have also been studied by investigating preferences for different settings. In a survey of 140 Norwegian adults, Hartig *et al.* [90] found that natural environments were generally preferred settings for restorative experiences when compared with urban settings. In an earlier study, Herzog *et al.* [66] compared three settings ranging from urban to natural to investigate which might promote restoration. Participants were given two scenarios to accomplish either psychological recovery or reflection and were then shown a series of slides of natural, sport/entertainment and urban settings which they had to rank to determine the suitability of the location in achieving the scenario goal. Natural settings showed uniformly high restorative potential in comparison with less natural settings. 

Studies have used self-report questionnaires and interviews with parents, guardians and teachers to gauge the effects of nature on children’s psychological well-being [62,91]. A study of children diagnosed with Attention Deficit Disorder (ADD) in the US tested whether indirect interactions with nature, such as exposure to nature during play, were related to attentional function [91]. Parents and carers of 96 children with ADD completed a questionnaire about the child’s attentional functioning after participating in leisure activities. Mean post-activity attentional functioning ratings were significantly higher for green outdoor activities as opposed to those in other settings, and the severity of ADD symptoms was significantly lower after playing in natural areas outdoors. It is unclear whether these psychological benefits are specific to children with ADD or applicable generally to all children. 

Three studies tested for the cognitive benefits of indirect interactions with nature such as viewing images [89] and incidental interactions such as encountering indoor foliage vegetation [58]. Berman *et al.* [89] found that the cognitive performance of students, as measured by three directed attention tasks, was significantly improved after viewing pictures of natural scenes rather than urban areas. A study in Japan found that indoor foliage vegetation had a similar beneficial effect on cognitive performance, but only for male participants [58]. The female participants showed higher task performance than the males, but the presence or configuration of indoor plants had no additional effect on their cognitive performance. However, a related study showed that cognitive performance declined with increased density of indoor plants [92]. It is possible that indoor environments may not require as high levels of directed attention in comparison with outdoor environments, thus the restorative influence of nature in indoor environments may have less impact on cognitive function. Further investigation in this area would be fruitful and future studies might usefully employ a randomised, controlled experimental design that accounts for variables such as gender, age, plant configurations, plant densities and species diversity. 

Incidental interactions with nature may positively influence the cognitive function of children. A longitudinal study in the United States correlated measures of vegetation around the home with measures of cognitive function to investigate the effect of moving from substandard low-income housing to new single family homes on 17 children [69]. There was a significant improvement in cognitive performance when the children moved to new homes surrounded by vegetation and natural elements. The author interprets this as evidence that presence of nature around the home is important for cognitive function in children [69]. However, no experimental control group was included, and although other possible explanatory variables (e.g., changes in housing quality) were analysed, it is unclear whether the presence of surrounding vegetation is the key variable influencing children’s cognitive performance, or what role other drivers such as the novelty of moving house or other features of the surrounding neighbourhood might play. Such multi-faceted analysis is of course difficult to achieve when one is relying on the goodwill of study participants.

In summary, the cognitive benefits of interacting with nature can be measured effectively using individual performance-based tests that are completed after interacting with nature in different settings. For this reason, objective and experimental research design has been employed by most studies investigating cognitive benefits. There is robust evidence to suggest that both intentional and indirect interactions with nature outdoors can improve cognitive function, although the evidence surrounding indoor interactions is more equivocal. Tightly controlled experiments are necessary to improve understanding in this area. As with psychological well-being benefits, much of the research into cognitive performance has made broad comparisons between green spaces and urban settings, and the specific effects of biodiversity on cognitive performance remain unknown.

#### 3.3.3. Physiological Benefits

Urban environments contain many potential stressors such as traffic, crime, dense crowds, and over-stimulation. There is evidence to suggest that interactions with nature may alleviate some of the negative physiological effects of such stressors. An experimental study in the Netherlands exposed participants to a stress-inducing activity and then measured cortisol levels following half an hour of either indoor reading or outdoor gardening [70]. While both activities led to decreases in cortisol levels, the decrease was significantly stronger for the gardeners, suggesting that gardening can relieve the physiological effects of stress [70]. However, because the control condition (indoor reading) is both passive and occurred in a non-natural setting, it is difficult to determine the relative contributions of the activity associated with gardening and the natural components of the environment in promoting stress reduction. 

In an experimental study in China, Yamaguchi *et al.* [71] measured salivary amylase activity as a physiological indicator of stress in healthy males before and after exercise in both a forest and an urban environment. Enzyme activity was significantly reduced after exercise in forest environments and the authors conclude that the physiological effects of stress are reduced in forest environments. A related study in Switzerland found that a decrease in stress-induced headaches, as measured by self-report surveys, was significantly related to physical activity in parks [37]. Interestingly the effect was consistent for park and forest environments, despite the very different biological characteristics of those environments.

Indirect interaction with nature such as a window view or a picture of nature may also reduce the physiological effects of stress. A study in Taiwan used electromyography and electroencephalography to test participants’ responses to photographs of office environments with different configurations of indoor plants and natural views [65]. Physiological condition improved when viewing a picture of an office with a view of nature and indoor plants, suggesting a reduction in the physiological effects of stress.

Other studies of physiological benefits have investigated the relationship between nature and healing. In a retrospective study of cholecystectomy patients in a hospital in Pennsylvania (USA), Ulrich [9] found that post-operative healing, as measured by length of hospital stay, took less time for patients in a hospital room with a window-view of nature in comparison with patients with a view of a brick wall. Patients with a view of trees also required fewer strong painkillers, received fewer negative evaluative comments from nurses and had fewer postsurgical complications. In another study, Bennett *et al.* [43] discovered that an outdoor therapeutic camping trip reduced the probability of patient relapse for recovering substance abusers.

Maas *et al.* [10] conducted a broad scale epidemiological study drawing together self-report perceived general health from 250,782 people in the Netherlands with national land cover data. Green space coverage within 1 km and 3 km of participants’ homes was positively related to perceived health. A related study from the UK employed land use datasets and mortality data to examine the gender differences in relationships between urban green space and health outcomes [11]. Male cardiovascular and respiratory disease mortality rates decreased with increasing green space, with no significant relationship for women [11]. By contrast, in an unrelated study, Ulrich [44] found that the positive physiological responses of exposure to nature, as measured by heart rate and alpha amplitude while viewing images of nature, were significantly stronger for women. Gender is clearly a factor that should be carefully considered when designing research programs.

Studies on the effects of indoor plants in office environments have shown that their presence can improve health [68] and reduce the occurrence of illness [15,35]. For example, Fjeld *et al.* [68] assessed the effect of office foliage plants on symptoms of discomfort for 51 healthy office workers at a Norwegian oil company over a two-year period. Participants completed a survey with questions relating to physical symptoms such as itchiness and coughing. The results showed that the presence of plants in offices correlated with a reduction in dry skin, hoarse throat, coughing and fatigue, suggesting that the introduction of foliage plants into an indoor environment may reduce symptoms of physical discomfort and improve health. Related studies on the effects of indoor vegetation have found that the number and presence of indoor plants in an office [35] and a classroom [15] reduce the occurrence and frequency of time off through ill health.

Overall, research to date has identified a broad range of physiological benefits from interacting with nature. There seems to be a good balance between experimental and correlative studies, and most have utilised both self-report and objective data. Whilst the evidence for some of the identified benefits is fairly strong, confounding factors such as gender and age have not always been incorporated into the research design. Most studies of the physiological benefits of interacting with nature are descriptive, and there is little evidence suggesting how these benefits are delivered. Despite the use of a variety of different natural settings, little explicit consideration has been given to the importance of biodiversity itself (rather than simply “green”) in delivering improved physical function or health.

#### 3.3.4. Social Benefits

Some of the negative impacts of urbanisation include individual isolation, lack of social support, interracial conflict and increased incidence of crime and violence [93]. Our review suggests that provision and access to green space within urban areas may ameliorate or even reverse some of these social challenges and ultimately increase social cohesion [4,72]. Interactions with nature can facilitate social interaction in adults and children [62], foster social empowerment [62,74], enhance interracial interaction [16] and promote social cohesion and support [73]. There is a significant decrease in crime rates and violent behaviour in urban areas with surrounding green space or vegetation, in comparison with urban areas with limited greenery [25,63]. 

Much of the reviewed literature for this type of benefit focused on the role of group-oriented conservation projects (e.g., tree planting) and community gardens in delivering social capital benefits. A study based in rural Australia discovered that residents of a community who were involved with a land management group had a greater sense of community cohesion and were more willing to work toward improving their community than non-participating individuals [59]. This provides some indication that volunteering in conservation groups might increase social capital within the wider community, although evidence from studies of other forms of intentional nature engagement activities suggests caution in interpretation. For example, Kingsley *et al.* [72] found that participants described social support, connections and social networking as positive elements of participating in a community garden. Whilst this might indicate that community gardens encourage wider social cohesion and cooperation [72], the authors acknowledge that the evidence from the study does not demonstrate that the social benefits extended beyond the garden community. Similarly, Shinew *et al.* [16] found that inter-racial interaction among community gardeners in North America was enhanced in community garden settings but did not extend into the wider community.

Other studies have focused on the role of urban green space and vegetation in reducing violence and crime in urban areas. Studying 98 urban apartment buildings in the city of Chicago, Illinois (USA), Kuo and Sullivan [25] found that crime rates, as measured by police crime reports, were significantly lower in buildings surrounded by higher vegetation density. Inclusion of confounding variables in the analysis produced the same results; vegetation density remained a consistent predictor of crime rates. In a related study of public housing residents in Chicago, Kuo and Sullivan [63] found that occurrences of violence and aggression, as measured through self-reports from residents, were also significantly lower in buildings with higher densities of surrounding vegetation. This study also investigated potential mechanisms behind the identified relationship and found that aggression was correlated with attentional function and, specifically, mental fatigue. Thus, a potential pathway is that urban vegetation restores attentional function, which reduces the occurrence of violence and aggression in urban public housing, indirectly contributing social benefits to the broader community. This fascinating Chicago-based work has not—to our knowledge—been replicated in any other study system; it would be fruitful to discover whether these results generalise to other locations, not least because there are myriad social benefits that stem from reduced violence and crime in urban settings.

The potential social benefits of nature interactions have also been studied in other sectors of society. Maller [62] conducted interviews with school principals and teachers in Melbourne, Australia to determine the perceived social benefits for children participating in nature-based activities. The identified benefits included empowerment and social engagement [62]. Whilst suggestive of social benefits, the results are based on the perceptions of the educators; development of longitudinal studies that follow students over time would provide further useful insight into these potential benefits for children.

#### 3.3.5. Spiritual Benefits

Research on the spiritual benefits of interacting with nature has been limited, with only three (5%) of the reviewed studies focusing specifically on these. Benefits included increased inspiration [42] and feelings of connectedness to a broader reality [47], both important for spiritual well-being [7]. It has been suggested that these types of benefits may also play an important role in positively influencing how people value natural environments [83] by inspiring a broader responsibility for the natural world [94].

Common elements among the multiple definitions of spiritual well-being include a sense of connectedness, a sense of purpose, a sense of awe and inspiration and faith in a larger reality [7,95,96]. These might or might not be connected to a specific religion *per se*; experiences of wilderness landscapes or specific natural features where the power of nature is apparent have been shown to invoke feelings of awe and inspiration. For example, a study of recreation programs in two separate wilderness areas in North America used on-site observations, journal analyses and interviews to examine wilderness experiences as a source of spiritual inspiration and well-being for women [42]. Participants reported on the expansiveness of landscapes and the power of nature as meaningful aspects of their wilderness experience. The authors interpreted this as evidence of spiritual inspiration and concluded that wilderness experiences are an opportunity for spiritual growth [42].

Viewing wildlife may invoke similar feelings of awe and inspiration, regardless of the environmental setting. In a study of tourists on two separate wildlife tours—in Spain and California—analysis of interviews and field journals revealed that viewing wildlife evoked a feeling of awe and wonder and created a temporal experience in which all concentration was focused on the moment [41]. This study suggests that wildlife-viewing events can promote a deep sense of well-being, leading to spiritual fulfillment.

One study investigated the occurrence of transcendent experiences in forest environments [47]. A transcendent experience is defined as one characterised by strong positive feelings of happiness and freedom, a sense of union with the universe or higher entity, absorption in the moment and a sense of timelessness [47]. This study, undertaken in Victoria, Australia, utilized a written questionnaire to prompt participants to recall a transcendent experience whilst in a forest environment. Participants included people who visit, live or work in forests. Two forms of transcendent experiences occurred in forests, one characterized by feelings of insignificance and the other by a sense of compatibility and familiarity. The authors interpret this as evidence of the spiritual value of forests [47].

Given the qualitative nature of the evidence, the lack of a single definition for spiritual well-being, small sample sizes and the limited number of studies, the evidence for spiritual benefits of nature experiences is not conclusive. There is a cultural bias towards Western society among the reviewed studies, despite the likelihood that the spiritual benefits of interacting with nature will vary significantly between cultures [76,97]. Also, the evidence for spiritual benefits is thus far largely limited to wilderness settings, which may have narrowed the range of benefits that have been studied. For example, feeding wildlife has been found to invoke a sense of belonging or connection to and, on a deeper level, a responsibility for the natural world [94]. In urban areas, where large natural areas are lacking, wildlife feeding may be an important mechanism for promoting spiritual well-being.

#### 3.3.6. Tangible Benefits

Interactions with nature can also produce tangible benefits. While large-scale industries such as agriculture and mining exemplify broad scale interactions that obtain tangible benefits through resource extraction or production, this review specifically focuses on individual interactions. Examples of these from amongst the 57 reviewed studies include food production from private domestic gardens [38,55], the impact of access to local green spaces on house sale prices [98,99,100], and the value of access to natural areas ascertained via willingness to pay for the experience [40].

In an early study on the benefits of gardening, a survey of gardeners in Michigan (USA) revealed that tangible benefits such as food production were rated as more important for plot gardeners than community or home gardeners [38]. The study also discovered that across all these gardeners other benefits, such as the gardening experience and sustained interest, were rated as more important than the tangible benefits. These results hint that while food production may be a tangible benefit, it may not be the primary motivation for gardening. In a recent study specifically focused on motivators for gardening amongst gardeners in Ohio (USA), producing food and herbs was one of the identified motivators for gardening but not the primary one [55]. Some disadvantaged neighbourhoods in developing countries largely depend on subsistence gardening as a source of food and income [101], thus cultural, regional and socio-economic differences may influence the relative importance of deriving tangible benefits from interacting with nature through gardening.

The economic value of access to natural areas can be calculated using hedonic valuation techniques, which estimate the influence of the natural area on the market price of another good, such as a house [98]. Morancho [98] used this technique to explain house prices in Castellon, Spain. Environmental variables included views of green space, distance from dwelling to nearest green space and the size of the nearest green space. Analyses revealed an inverse relationship between house prices and distance to nearest green space and that proximity is more important than size of the nearest green space [98].

Using the same hedonic technique, Bolitzer and Netusil [75] analysed sale price data for houses in Portland, Oregon (USA). Proximity to green space had a positive effect on home sale prices, but these effects were only significant for publicly accessible green spaces such as public parks or golf courses. While agreeing with Morancho’s [98] findings, these results highlight that the type of urban green space can be important in addition to its proximity. This may be because people place value on green space they can access, suggesting that inaccessible private green space may be of less value than a publicly available park, although further research on this potential distinction would be fruitful. In a related study, Pearson *et al.* [50] used the hedonic pricing technique to estimate the impact of the headland section of Noosa National Park, Australia, on land values in areas surrounding the park. In contrast to the other studies [75,98], while views of the National park increased property value, proximity had no significant effect [50]. Moreover, working in Baltimore, Maryland (USA), Troy and Grove [99] identified a negative effect of proximity to green space in neighbourhoods where crime rates were above a certain threshold, suggesting that green space can be viewed as a liability in some situations.

The economic value of recreation in natural environments can also be estimated at an individual level. In a study of three public beaches in South Carolina (USA), Oh *et al.* [40] surveyed 925 non-resident visitors to public beaches and found that participants were willing to pay on average an extra $6.60 per day for additional public beach access points in South Carolina. The identified willingness to pay an increased entry fee may be indicative of the economic value that people place on the benefits of visiting a beach.

## 4. Conclusions

Our review has identified a typology of three categories of nature-interaction—indirect, incidental and intentional—as well as six key types of benefits that may be experienced from a variety of natural settings. All of the reviewed studies were undertaken in developed countries, mostly Western societies, and there is a clear bias in the literature with respect to cultural and socio-economic differences between geographic regions. It is thus difficult to determine which of the reported benefits of interacting with nature are universal, and which are culturally specific. While it has been hypothesized that humans have an instinctive connection with nature [3], this connection may manifest itself differently in different cultures owing to different value systems and attachments to natural areas. A mix of theoretical and empirical work may well be needed to further examine this important dimension of the benefits of interaction with nature.

The body of literature is broad and spans several disciplines although the majority has been conducted from within the social sciences. The noticeable lack of contribution from the environmental and biological sciences has meant that we know little about which specific ecological features of the environment might be important for delivering a beneficial response. The sophisticated tools available within ecology for measuring characteristics of the biological component of landscapes, such as species richness, vegetation structure and community composition, could provide fruitful insight into this issue. For example, what type of vegetation structure is needed, and how many species should be planted to maximise the well-being value of an urban green space? To remedy this, we encourage further engagement by ecologists to investigate the extent to which the biological richness of a landscape, as opposed to an often loosely defined “nature”, plays an important role in enhancing beneficial interactions.

Overall, there is good evidence to suggest that natural settings can have multiple beneficial effects. It was clear from the reviewed studies that some types of benefits have been much more heavily studied than others; psychological, cognitive and physiological benefits featured most prominently with fewer studies of social, spiritual and tangible benefits. Given the possible wider societal benefits from increased social cohesion [4] or the role that spiritual well-being might play in influencing the way people value nature [94], the latter benefits deserve considered attention in future research.

When drawing conclusions from the existing literature, this review suggests caution is appropriate. There were several general methodological limitations that recurred throughout the body of reviewed literature. Firstly, much of the evidence has been derived from self-report questionnaires, particularly in the studies focused on psychological and social benefits. Secondly, sampling bias may have influenced results in some cases, especially for studies that recruited participants *in situ*. Thirdly, the reviewed studies were generally conducted over relatively short time frames leaving our understanding of the long-term benefits of interacting with nature unstudied. Lastly, many experimental studies did not include an appropriate control group therefore confounding variables such as age, gender and personal values may have influenced the results.

The summative insight across these studies provides important indication of the potential range of benefits yet there remains the possibility that some or many of the widely accepted benefits of interacting with nature are actually not causally related to nature itself. For example, while the psychological benefits associated with exercising in natural areas have received considerable focus, there is limited evidence to suggest that these interactions have positive psychological and physiological effects. It is clear, though, that whilst exercising in natural environments may not deliver psychological and physiological benefits, natural areas may be important for facilitating exercise, particularly in urban areas. Similarly, there is strong evidence to suggest that natural settings, such as community gardens, can be important for facilitating social contact, though it is unclear whether collaborative activities in these natural settings can actually increase social capital in the wider community.

Interactions with nature can positively influence behaviour, academic performance and social skills in children [62,69], something that could be reflected in school curricula (e.g., environmental experience components) and could have broader benefits, such as reducing bullying in schools. Much of the evidence is based on self-reported perceptions of parents and guardians, and the majority of these studies have been undertaken over relatively short timeframes. For example, while childhood interactions with nature may influence attitudes towards the environment in later life [79], which could have positive implications for future conservation efforts, longitudinal studies will be necessary to more fully evaluate the development of environmental values and behaviours from childhood.

Understanding the benefits of interacting with nature is also important from a sustainability perspective. Unsustainable exploitation of natural resources has resulted in the loss and degradation of species and ecosystems worldwide [102]. The natural systems that have sustained human livelihoods throughout history have been severely impacted, consequently human quality of life has been inadvertently threatened. The solutions to the current biodiversity crisis are complex and will depend on broad-scale conservation efforts, effective landscape management and innovative urban planning. Strong public support will be necessary to encourage governments to implement effective conservation policies [102], and positive interactions with nature might be important for influencing an individual’s sympathy for conservation goals [83]. However, opportunities for these interactions are being reduced as urbanisation increases and the majority of the human population now resides in urban areas [83,103]. Therefore, protecting and enhancing biodiversity in these urban areas could be critical for achieving conservation objectives and maintaining human quality of life during this period of major global change.

Overall, this literature review has documented a broad range of the benefits of interacting with nature. It has been shown that interactions with nature can deliver a range of psychological well-being, cognitive, physiological, social, tangible and spiritual benefits and that access to green space and natural areas is important for facilitating activities that are beneficial for human well-being. However, because the evidence is mostly descriptive, little is known about the mechanisms that are important for delivering these benefits and so key questions still remain. What characteristics of natural settings (e.g., biodiversity, level of disturbance, proximity, accessibility) are important for triggering a beneficial interaction? How do these characteristics vary in importance between different cultures, geographic regions and socio-economic groups? These are important directions for future research if we are to make effective, informed decisions regarding the best ways to maximise opportunities for people to interact with nature in a rapidly urbanising world.
